# Modulation of Apoptotic Cell Death and Neuroprotective Effects of Glutathione—L-Dopa Codrug Against H_2_O_2_-Induced Cellular Toxicity

**DOI:** 10.3390/antiox8080319

**Published:** 2019-08-19

**Authors:** Sara Franceschelli, Paola Lanuti, Alessio Ferrone, Daniela Maria Pia Gatta, Lorenza Speranza, Mirko Pesce, Alfredo Grilli, Ivana Cacciatore, Emanuela Ricciotti, Antonio Di Stefano, Sebastiano Miscia, Mario Felaco, Antonia Patruno

**Affiliations:** 1 Department of Psychological, Health and Territorial Sciences, University “G. D’Annunzio”, 66100 Chieti-Pescara, Italy; 2 Department of Medicine and Science of Aging, University “G. D’Annunzio”, 66100 Chieti-Pescara, Italy; 3 Department of Pharmacy, University “Gabriele D’Annunzio” of Chieti-Pescara, 66100 Chieti-Pescara, Italy; 4 Department of Systems Pharmacology and Translational Therapeutics, University of Pennsylvania, Philadelphia, PA 19104, USA

**Keywords:** L-Dopa, glutathione, SH-SY5Y cell line, U-937 cell line, Parkinson’s disease, ROS, apoptosis

## Abstract

The L-3,4-dihydroxyphenylalanine (LD) is the gold standard drug currently used to manage Parkinson’s disease (PD) and to control its symptoms. However, LD could cause disease neurotoxicity due to the generation of pro-oxidant intermediates deriving from its autoxidation. In order to overcome this limitation, we have conjugated LD to the natural antioxidant glutathione (GSH) to form a codrug (GSH-LD). Here we investigated the effect of GSH-LD on H_2_O_2_-induced cellular toxicity in undifferentiated and differentiated lymphoma U-937 and dopaminergic neuroblastoma SH-SY5Y cell lines, used respectively as models to study the involvement of macrophages/microglia and dopaminergic neurons in PD. We analyzed the effect of GSH-LD on apoptosis and cellular oxidative stress, both considered strategic targets for the prevention and treatment of neurodegenerative diseases. Compared to LD and GSH, GSH-LD had a stronger effect in preventing hydrogen peroxide (H_2_O_2_) induced apoptosis in both cell lines. Moreover, GSH-LD was able to preserve cell viability, cellular redox status, gluthation metabolism and prevent reactive oxygen species (ROS) formation, in a phosphinositide 3-kinase (PI3K)/kinase B (Akt)-dependent manner, in a neurotoxicity cellular model. Our findings indicate that the GSH-LD codrug offers advantages deriving from the additive effect of LD and GSH and it could represent a promising candidate for PD treatment.

## 1. Introduction

Parkinson’s disease (PD) is a neurodegenerative disorder characterized by the progressive loss of dopaminergic neurons mainly in the substantia nigra. Therefore, in PD patients there is decreased dopamine (DA) nigrostriatal availability associated with Lewis bodies formation composed by fibrillar α-sinuclein [1]. Administration of L-3,4-dihydroxyphenylalanine (LD) represents the main pharmacotherapeutic treatment for PD. Recent studies have indicated a possible involvement of LD to disease progression due to the generation of pro-oxidant intermediates deriving from its autoxidation [2]. For these reasons, new therapeutic strategies based on DA replacement therapy, are needed to reduce the inexorable progression of PD. DA metabolism, mitochondrial dysfunction, and neuro-inflammation, which cause free radical/reactive oxygen species (ROS) generation in dopaminergic neurons and consequent oxidative stress-triggered neuronal death in the substantia nigra, have been greatly implicated in PD pathogenesis [3,4]. Several studies have demonstrated that ROS generation and the resulting changes in cellular redox status can induce signal transduction pathways that are responsible for the apoptotic process [5]. Recently, the activation of the apoptotic cascade in dopaminergic neurons of parkinsonian substantia nigra was observed, and this was proposed as a cause of cell death in PD. It has been suggested that apoptosis in dopaminergic neurons leads to activation of microglia and then to the onset of an inflammatory processes which determines, through a vicious cycle, the progression of PD disease through an acceleration of neurodegeneration [6]. Several pharmacological strategies have attempted to interfere with the apoptotic process in order to protect the functionality of dopaminergic neurons that are compromised in PD. It was found that PD patients are characterized not only by decreased levels of dopamine but also by reduced levels of the antioxidant-redox modulators of the brain such as glutathione (GSH) [7]. The nervous system (NS) is vulnerable to ROS formation and GSH deficiency since they both are important factors contributing to oxidative stress. Thus, restoration of normal GSH levels in the NS, aiming to protect DA neurons from oxidative stress injuries, may represent a potential alternative treatment for PD [8].

**Figure 1:**
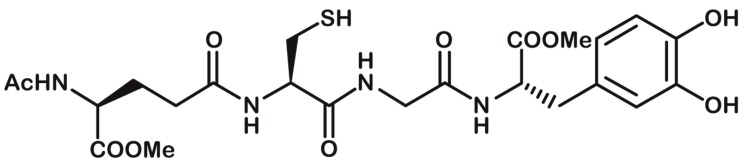
Chemical structure of glutathione-L-3,4-dihydroxyphenylalanine (GSH-LD) codrug.

We previously reported the antioxidant efficacy of a codrug (GSH-LD), obtained by linking covalent LD via an amide bond with GSH in the C- and N-terminal position, respectively [9] (Figure 1).

In the current study we aimed to explore whether this codrug could protect against the apoptotic consequences of the H_2_O_2_-induced oxidative stress and thus represent a potential candidate for the treatment and/or prevention of neurodegenerative diseases. The human neuroblastoma SH-SY5Y cell line has been extensively used to study dopaminergic neuron-like behavior in response to toxic insults in PD. Following stimulation with *cis*-retinoic acid (RA), proliferating SH-SY5Y cells can be differentiated into non-proliferating neuronal cells with a dopaminergic phenotype. Both undifferentiated and RA-differentiated SH-SY5Y cells have been widely used as a cellular model of PD [10]. Over the last decades, growing lines of evidence support the existence of a connection between the central nervous system (CNS) and the immune system as a unique bidirectional communication system [11]. Indeed, in PD patients, alterations in circulating monocytes, in terms of their transcriptome profile, subset composition, and function have been reported, indicating a role for the innate immune system in dopaminergic cell death [12]. Total monocyte numbers and ROS production are increased, while proteasome activity and dopamine transporter expression of monocytes are decreased in PD. These abnormalities suggest that monocyte function is impaired in the peripheral blood in PD [13]. In order to take in account for the contribution of resident-peripheral immune cells in the development of neurodegenerative disorders, we have expanded the study of GSH-LD on H_2_O_2_-induced cellular toxicity in undifferentiated and differentiated human monocytic cell lines (U937). Moreover, we have investigated the effect of GSH-LD in a neurotoxicity model that mimics the interplay between microglia and neurons.

## 2. Materials and Methods

### 2.1. Cell Culture and Treatment

Undifferentiated human monocytic cell line U937 (U_U937_) (CRL-1593.2) and undifferentiated human neuroblastoma cell line SH-SY5Y (U_SH-SY5Y_) (CRL-2266) (American Type Culture Collection, Manassas, VA, USA) were cultured following the manufacturing protocol. For differentiation, U937 cells were grown overnight on a 6-well plate at a density of 8 × 10^5^ cells per well. Differentiate U937 cells (D_U937_) were then incubated with PMA (10 ng/mL) for 24 h and washed with PBS to remove non-adherent cells.

For differentiation, SH-SY5Y cells were seeded at an initial density of 10^4^ cells/cm^2^ in culture dishes and incubated for five days in the medium with 10 *u*M 13-*cis*-RA (Sigma). The cells were then incubated in serum-free Dulbecco’s Modified Eagle’s Medium (DMEM) with 100 ng/mL Brain-derived neurotrophic factor (BDNF) (Alomone Labs, Har Botzvim Hi-Tech Park, Jerusalem, Israel) for an additional five days to generate the differentiated SH-SY5Y cells (D_SH-SY5Y_).

At subconfluence (70–80%), U937 and SH-SY5Y cells cultured in 24-well plates were pre-exposed to test compounds for 1 h and then stimulated with H_2_O_2_ 500 µM for 4 h.

LD-GSH was dissolved in dimethyl sulfoxide (DMSO) and diluted with the medium. The final concentration of DMSO in the medium was 0.5%. MTT (3-(4,5-Dimethyl-2-thiazolyl)-2,5-diphenyl-2H-tetrazolium bromide) assay for cell viability was performed as described previously [14].

### 2.2. Effects of Drugs on the Cytotoxicity of U937 Cell Supernatants

The cytotoxicity experiments were performed using D_U937_ cells seeded into 24-well plates at a concentration of 2 × 10^6^ cells per well in 0.8 mL of RPMI 1640 medium containing 5% FBS (Fetal bovine serum) as previously reported [15]. The cells were incubated with an activating stimulus (H_2_O_2_ 500 µM). After 4 h incubation, 0.5 mL of cell-free supernatant was transferred to each well containing D_SH-SY5Y_ cells plated earlier at a concentration of 4 × 10^5^ cell mL^−1^.

After 24 h of incubation, the cells were sampled for an MTT assay. The GSH-LD compound was added to D_SH-SY5Y_ cells 1 h before transferring D_U937_ cell supernatants to wells with neuronal cells.

### 2.3. Cell Apoptosis Assessment

Following treatment with LD, GSH-LD and GSH (1, 10 and 100 μM) for 4 h, the H_2_O_2_ (500 μM)-treated cells were stained with Annexin V-propium iodide (Thermo Fisher Scientific, Waltham, MA, USA) and apoptosis rates were analyzed using a FACScanto II flow cytometer (BD, Becton-Dickinson Biosciences, San Jose, CA, USA). Data were analyzed with FlowJo software v8.8.6 (TreeStar, Ashland, OR, USA) and Flow Cytometry (FCS) Express 5 Software (De Novo Software, Glendale, CA, USA).

### 2.4. Western Blot Analysis

Western blot analysis was performed as described previously [16] using the following antibodies against rabbit polyclonal Bcl-2 (Sc-492; 1:500), rabbit polyclonal cleaved caspase-3 (Sc-22171-R; 1:400), rabbit polyclonal Bax (Sc-493; 1:500), rabbit polyclonal pAkT (sc–135650; 1:1000), rabbit polyclonal p-PI3-kinase (sc-12929; 1:500) and mouse monoclonal β-actin (Santa Cruz Biotechnology, Inc., Dallas, TX, USA). The blots were then incubated for 1 h at room temperature with goat anti-mouse secondary antibody (Sc-2005; 1:2000; Santa Cruz Biotechnology) or polyclonal goat anti-rabbit secondary antibody (Sc-66931; 1:5000; Santa Cruz Biotechnology).

To verify if the U937-PMA driven differentiation was also associated with the expression of macrophage-selective markers, we performed Western blot analyses with the following monoclonal antibodies: anti-CD206 (monoclonal, 1:1000, Santa Cruz), anti-CD14 (monoclonal, Santa Cruz). The differentiation of SH-SY5Y cells was detected using the following antibodies: anti-Nestin (monoclonal, 1:1000, Santa Cruz), rabbit anti-MAP2 (policlonal, 1:1000, Millipore) (Figure S1).

### 2.5. Caspase 3 Assay

Caspase-3 (EC 3.4.22.56) activity was determined using the fluorogenic substrate Ac-DEVD-pNA (Ac-Asp-Glu-Val-Asp-7-amino-4-methylcoumarin; Enzo Life Sciences LTD, Exeter, UK). Briefly, cells were centrifuged at 1000× *g* for 5 min at 4 °C and lysed in lysis buffer (50 mM Tris pH 7.5, 150 mM NaCl, 5 mM EDTA (ethylenediaminetetraacetatedihydrate), and 0.2% Nonidet P-40). After centrifugation at 17,000× *g* for 10 min at 4 °C, the reaction buffer containing HEPES (4-(2-Hydroxyethyl) piperazine-1-ethanesulfonic acid)) (10 mM, pH 7.5), NaCl (50 mM), MgCl_2_ (5 mM), DTT (Dithiothreitol) (2.5 mM), and EDTA (1 mM) was added. Samples were incubated with substrate (50 μM) and fluorescence (substrate turnover) was determined by excitation at 360 nm and emission at 460 nm in a 96-well microplate reader (model 550; Bio-Rad Laboratories, Inc., Hercules, CA, USA). The rate of substrate hydrolysis was monitored at 37 °C.

### 2.6. ROS Detection

ROS production through flow cytometry analysis was performed as previously described [17]. Cells (1 × 10^4^ cells/well) were seeded in a 96-well dark plate and incubated for 40 min at 37 °C with freshly prepared DCFDA (2’,7’-dichlorofluorescin diacetate; D6883, Sigma Aldrich) reconstituted in DMSO at a final concentration of 25 μM. The cells were washed twice with PBS cold and centrifuged at 400× *g*. The cell pellets were then dissolved in Triton X-100 (1%). The fluorescence generated by DCFDA oxidized to a 2′,7′-dichlorofluorescein (DCF) was detected at a wavelength of 480 (excitation)/530 nm (emission) by FlexStation fluorescence plate reader (Molecular Devices, Sunnyvale, CA, USA). An NBT (nitroblue tetrazolium) assay was performed as previously described [18]. Briefly, the following were added to each well of a 96-well plate: 100 μL potassium phosphate buffer (pH 7.8, 50 mM), catalase (5 μL), NBT (25 μM, 5.6 × 10^−9^ M), xanthine (50 μL, 0.1 mM), xanthine oxidase (50 μL, 0.1 mM) and GSH-LD 1 μM. Following the addition of NBT the plates were allowed to stand at room temperature for 1 h until the blue color had developed and the absorbance was measured at 620 nm using a microplate reader (Bio-Rad Laboratories, Inc., Hercules, CA, USA).

### 2.7. Activity of Antioxidant Enzymes

Superoxide dismutase (SOD, EC 1.15.1.1) activity was performed as previously described [19] using an assay mixture containing sodium carbonate buffer (50 mM, pH 10) epinephrine (0.1 mM), and fresh cell lysate (containing 25 μg of protein) in a final volume of 200 μL [20]. Percentage inhibition values were converted into activities by using a purified Cu, Zn bovine SOD as standard. One unit of SOD is the amount of enzyme required to halve the rate of substrate auto-oxidation.

Catalase (CAT, EC 1.11.1.6), glutathione peroxidase (GPx, EC 1.11.1.9) and glutathione reductase (GR, EC 1.8.1.7) activity was measured as described previously [20]. CAT activity was measured spectrophotometrically monitoring the decomposition of H_2_O_2_ at 240 nm. The assay mixture in a final volume of 3 mL, contained potassium phosphate buffer (10 mM), H_2_O_2_ (10 mM) and 5 μg of enzymatic extract protein. CAT units were defined as 1 μmole H_2_O_2_ decomposed/min at 25 °C.

The quantification of the GPx activity was measured using H_2_O_2_ (0.25 mM) as a substrate. The oxidation of NADPH (Nicotinamide adenine dinucleotide 2’-phosphate reduced) was observed at 25 °C on a spectrophotometer at 340 nm. One unit was defined as 1 mmol of GSH oxidized/min.

The GR activity was specrophotometrically monitored at 340 nm and 25 °C. The assay mixture in a final volume of 1 mL contained potassium phosphate buffer (0.1 mM, pH 7.4), of EDTA (1 mM), of Glutathione disulfide (GSSG) (1 mM) (Sigma, St. Louis, MI, USA), NADPH (0.16 mM) (Sigma, St. Louis, Missouri, USA) and 1–30 micrograms of cellular protein. One unit of enzyme activity was defined as 1 mmol of NADPH oxidized/min at 25 °C.

### 2.8. GSH Assay

For reduced glutathione detection, cells were washed twice with Ca^2+^/Mg^2+^ free PBS and resuspended in a buffer containing ascorbic acid (10 mM), HCl (20 mM), trichloroacetic acid (5%) and diethylene triamine pentaacetic acid (5 mM) (Sigma Aldrich, US, Canada). After centrifugation (12,000× *g*), the supernatant was neutralised with potassium phosphate buffer (1 M; pH 7.0) and shared in two aliquots. One of these had N-ethylmaleimide (0.45 mM) added. Then potassium phosphate buffer (0.1 M, pH 7.0) was added to both samples, which then had o-phthalaldehyde (0.05%) added. The fluorescence was measured using a Plate Reader (Bio-Rad, Hercules, CA, USA) at an excitation wavelength of 355 nm and an emission wavelength of 430 nm. Reduced glutathione level in a sample was displayed as difference between fluorescence values in non-derivatised and N-ethylmaleimide-derivatised sample aliquots.

### 2.9. Statistical Analysis

All results were expressed as mean ± standard deviation. Statistical significance was calculated by one-way analysis of variance (ANOVA), and *p* < 0.05 values were considered statistically significant.

## 3. Results

### 3.1. Effect of GSH-LD on Cell Viability

**Figure 2:**
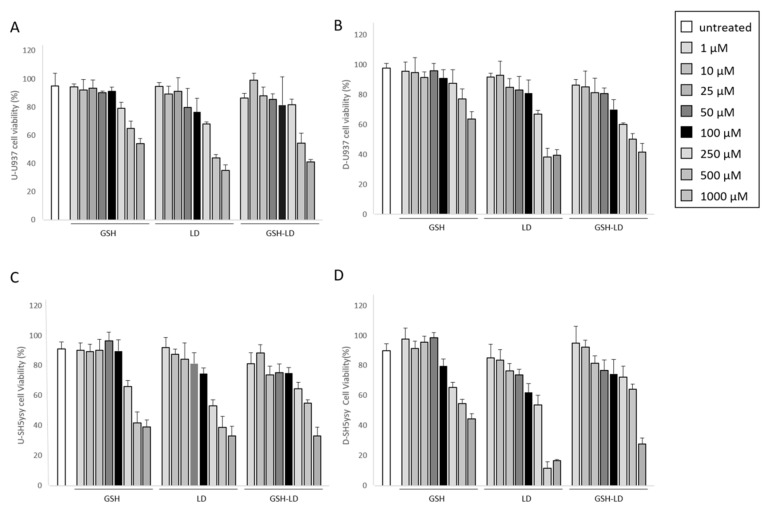
Effect of compounds on cell viability. The MTT (3-(4,5-Dimethyl-2-thiazolyl)-2,5-diphenyl-2H-tetrazolium bromide) cell viability of GSH, LD and GSH-LD (1, 10, 50, 100, 250, 500 and 1000 µM), on (**A**) undifferentiated U937 (U_U937_) cells; (**B**) differentiated U937 (D_U937_) cells; (**C**) undifferentiated SH-SY5Y (U_SH-SY5Y_) and (**D**) differentiated SH-SY5Y (D_SH-SY5Y_) using an incubation time of 24 h.

The cytotoxic effect of GSH-LD co-drug and of the single compounds LD and GSH on U_U937_, D_U937_, U_SH-SY5Y_ and D_SH-SY5Y_ cells was assessed by the MTT assay after 24 h of treatment. The viability of U_U937_, D_U937_, U_SH-SY5Y_ and D_SH-SY5Y_ cells did not change with compound concentrations ranging from 1 to 100 μM. Conversely, compound-mediated cellular toxicity was observed at 250 and 1000 μM (Figure 2). Based on these results, the compound concentrations of 1, 10 and 100 μM for both U937 and SH-SY5Y cells were used for subsequent experiments. The cyto-protective effect of GSH-LD against oxidative stress was assayed using a H_2_O_2_ induced cytotoxicity model for the generation of exogenous free radicals through the activation of caspase-3 [21,22]. A cell viability assay was performed to identify the suitable concentration (500 μM) and incubation time (4 h) for H_2_O_2_ cyto-toxic effect (data not shown).

### 3.2. Effect of GSH-LD on H_2_O_2_-Induced Apoptosis in Undifferentiated and Differentiated U937 Cells

**Figure 3:**
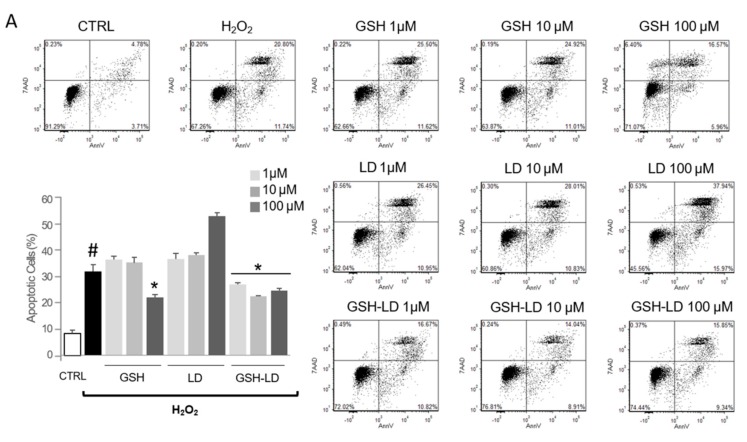

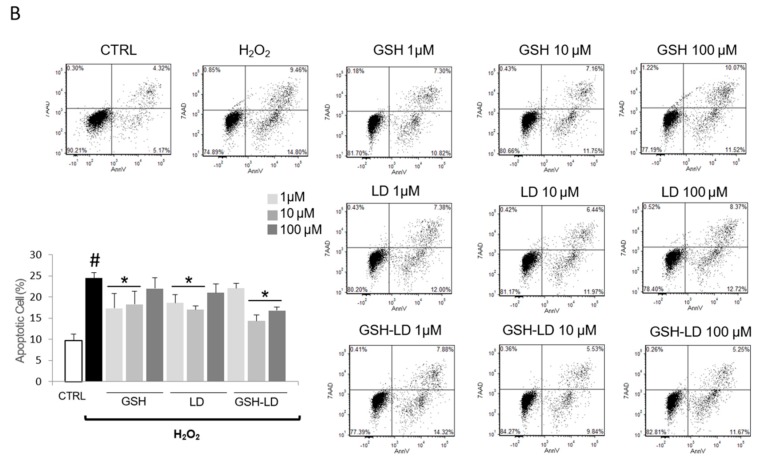
Effects of compounds on H_2_O_2_-induced apoptosis in a monocytic cell line. Effects of GSH, LD and GSH-LD on H_2_O_2_-induced apoptosis of U_U937_ (**A**) and D_U937_ (**B**). Cells were treated with different concentrations of compounds (1, 10 and 100 μM) for 1 h, and then exposed to H_2_O_2_ (500 μM) for 4 h. An Annexin V assay was used for apoptosis detection. Data is presented as the mean ± standard deviation (*n* = 6). ^#^ *p* < 0.01, vs. control (CTRL); * *p* < 0.01 vs. H_2_O_2_-treated cells.

The treatment with H_2_O_2_ caused a significant increase in apoptotic rate in both U_U937_ and D_U937_ cells as assessed by Annexin V/propidium iodide staining (Figure 3A,B). The pre-treatment with GSH or LD has no effect on H_2_O_2_-induced apoptosis in U_U937_ (except for GSH at 100 µM). In D_U937_, the pre-treatment with GSH or LD, caused a significant reduction of the number of apoptotic cells at concentrations of 1 and 10 µM. Contrary to this, the pre-treatment with GSH-LD caused a significant reduction of the number of apoptotic cells in both in U_U937_ and D_U937_, although a significant reduction of apoptotic rate in D_U937_ was achieved only using 10 µM of GSH-LD (Figure 3A,B); this concentration was used for all subsequent experiments.

**Figure 4:**
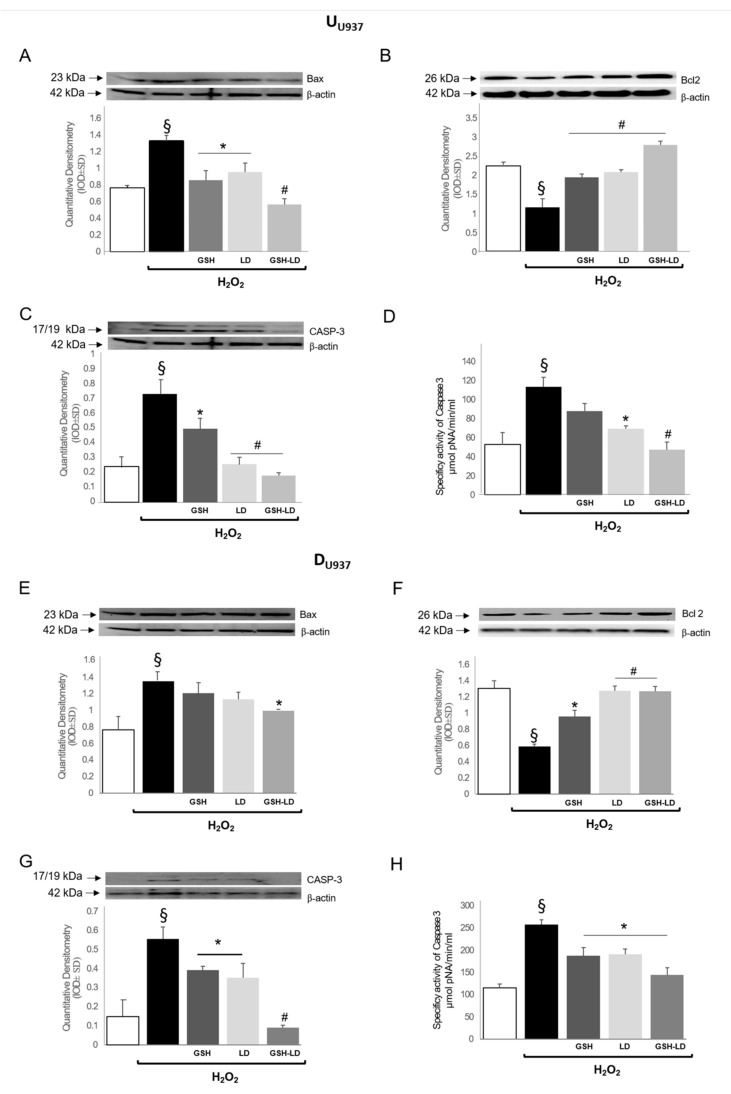
Effects of compounds on expression levels of apoptosis related proteins in monocytic cell line. Effect of GSH, LD and GSH-LD (10 μM) on the expression levels of Bax (**A**,**E**) and Bcl-2 (**B**,**F**) and on caspase 3 expression and fluorescent activity (**C**,**D**,**G**,**H**), on H_2_O_2_-treated U_U937_ (upper) and D_U937_ (lower) cells. ß-actin was used as internal standard. Data is presented as the mean ± SD (*n* = 6), ^§^ *p* < 0.05 vs. CTRL; ^#^ *p* < 0.01, * *p* < 0.05 vs. H_2_O_2_-treated cells.

The effect of GSH-LD on apoptosis in U_U937_ and D_U937_ cells was further analyzed by detecting the protein expression of caspase 3, Bax and Bcl-2 by Western blot analyses and by measuring caspase-3 activity by a fluorescent assay. The treatment with H_2_O_2_ acts as stress sensors of the cell and triggers the expression of pro-apoptotic proteins Bax (Figure 4A,E) and caspase 3 (Figure 4C,G) in both in U_U937_ and D_U937_ cells. All three pre-treatments caused a significant reduction of the expression of the apoptotic proteins Bax and Caspase-3 and an increase of the expression of the antiapoptotic protein Bcl-2 in U_U937_ cells (Figure 4B), and both LD and GSH-LD also caused a reduction of caspase-3 activity in the same cells (Figure 4D). The effect of GSH-LD on pro-apoptotic and anti-apoptotic proteins and on caspase-3 activity was stronger compared to the effect of LD or GSH alone. Similarly, all three pre-treatments caused a significant reduction of the expression of Caspase-3 (Figure 4G) and an increase of the expression Bcl-2 in D_U937_ cells (Figure 4F), while only GSH-LD was able to reduce the expression of Bax (Figure 4E). All three pre-treatments caused a reduction of caspase-3 activity in the same cells, with a stronger effect obtained with GSH-LD (Figure 4H). These data indicate that GSH-LD has an antiapoptotic effect in H_2_O_2_ treated U_U937_ and D_U937_ cells.

In conclusion, in undifferentiated monocytes, GSH-LD co-drug treatment showed more capacity to inactivate caspase 3 and then to reduce the apoptotic effect at lower concentration compared to GSH alone.

### 3.3. Effect of GSH-LD on H_2_O_2_-Induced Apoptosis in Undifferentiated and Differentiated SH-SY5Y Cells

**Figure 5:**
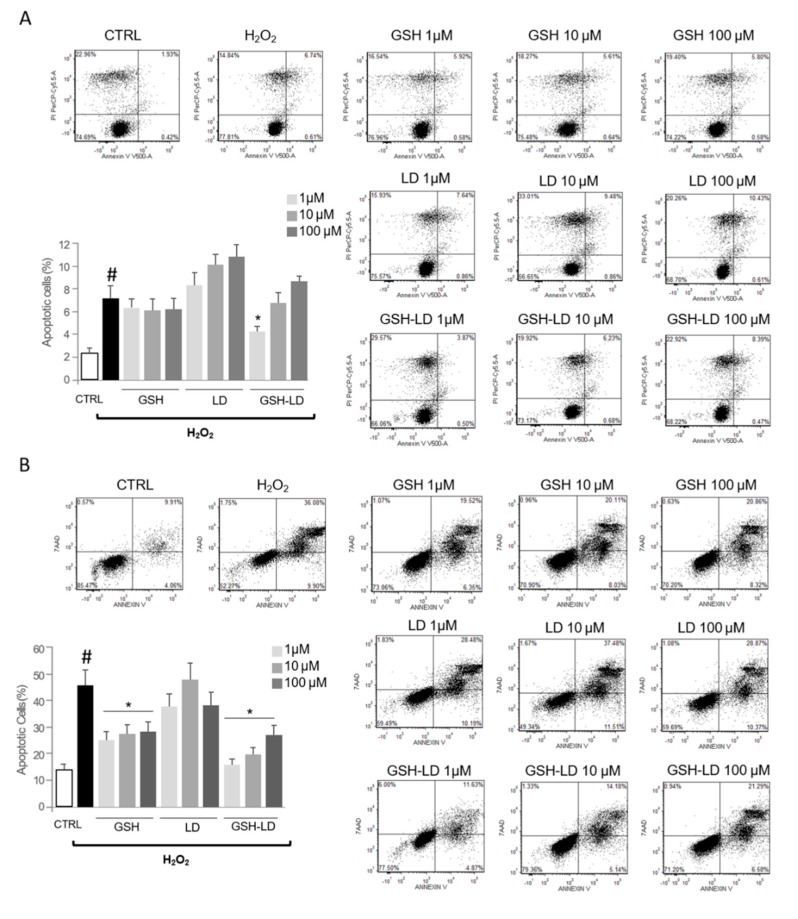
Effects of compounds on H_2_O_2_-induced apoptosis in neuroblastoma cell line. Effects of GSH, LD and GSH-LD on H_2_O_2_-induced apoptosis on (**A**) U_SH-SY5Y_ and (**B**) D_SH-SY5Y_ cells. Cells were treated with different concentrations of compounds (1, 10 and 100 μM) for 1 h, and then exposed to H_2_O_2_ (500 μM) for 4 h. An Annexin V assay was used for apoptosis detection. Data are presented as the mean ± standard deviation (*n* = 6). ^#^ *p* < 0.01 vs. CTRL; * *p* < 0.01 vs. H_2_O_2_-treated cells.

The treatment with H_2_O_2_ caused a significant increase in apoptotic rate in both U_SH-SY5Y_ and D_SH-SY5Y_ cells as assessed by Annexin V/propidium iodide staining (Figure 5). Firstly, the pre-treatment with GSH turns out to be effective only on differentiated cells. Secondly, the results from Annexin V/PI staining, indicated that the apoptotic rates, were higher in both in U_SH-SY5Y_ and D_SH-SY5Y_ cells co-treated with H_2_O_2_ and LD compared to the H_2_O_2_-treated cells. The pre-treatment with GSH or LD has no effect or increase the number of apoptotic cells in H_2_O_2_-treated U_SH-SY5Y_ cells. On the contrary, GSH-LD caused a reduction of apoptosis in the same cells, although the reduction was significant only when GSH-LD was used at 1 μM (Figure 5A). The pre-treatment with GSH or GSH-LD caused a reduction of the number of apoptotic cells in H_2_O_2_-treated D_SH-SY5Y_ cells, while LD had an opposite effect (Figure 5B). GSH-LD has a stronger anti-apoptotic effect compared to GSH in H_2_O_2_-treated U_SH-SY5Y_ and D_SH-SY5Y_ cells. Since GSH-LD showed an anti-apoptotic effect already at 1 μM in U_SH-SY5Y_ and D_SH-SY5Y_ cells, this concentration was used for all subsequent experiments.

**Figure 6:**
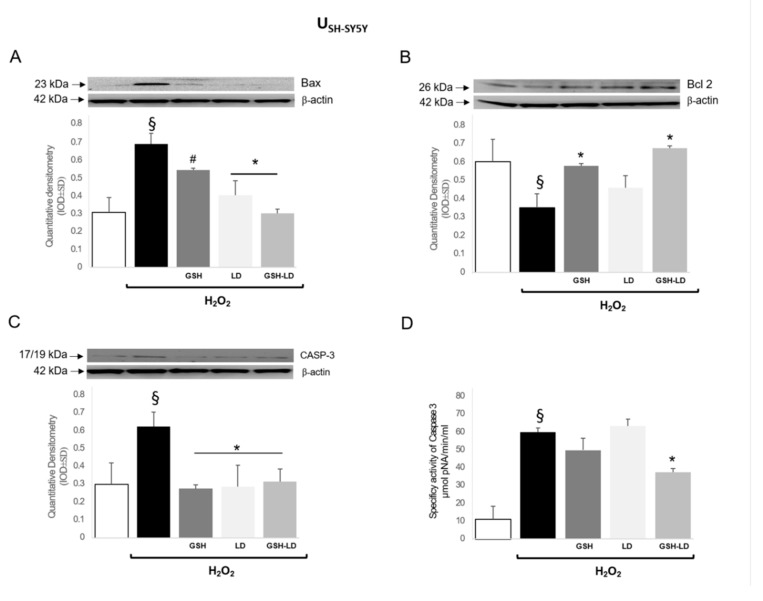

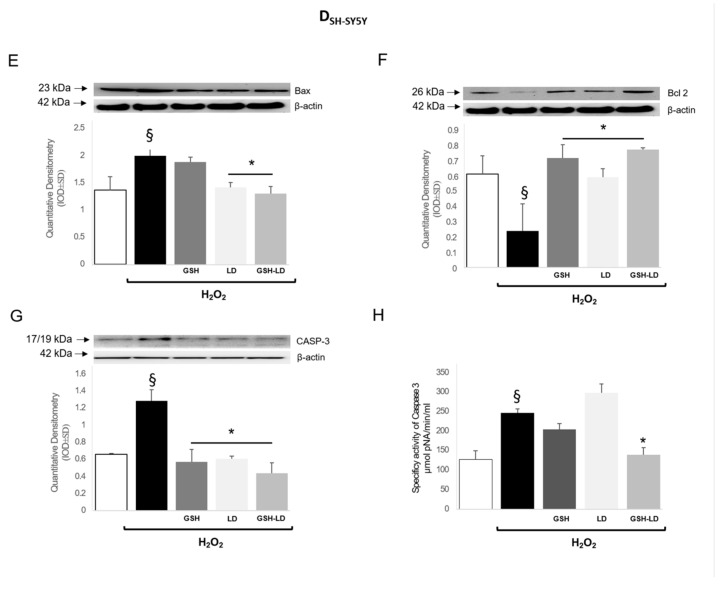
Effects of compounds on expression levels of apoptosis related proteins in the neuroblastoma cell line. Effect of GSH, LD and GSH-LD (1 μM), on the expression levels of Bax (**A**,**E**) and Bcl-2 (**B**,**F**) and on caspase 3 expression and fluorescent activity (**C**,**D**,**G**,**H**), on H_2_O_2_-treated U_SH-SY5Y_ (upper) and D_SH-SY5Y_ (lower) cells. ß-actin was used as internal standard. Data are presented as the mean ± SD (*n* = 6), ^§^ *p* < 0.05 vs. CTRL; ^#^ *p* < 0.05 and * *p* < 0.01 vs. H_2_O_2_-treated cells.

The treatment with H_2_O_2_ induced the expression of pro-apoptotic proteins Bax (Figure 6A,E) and caspase 3 (Figure 6C,G) and reduced the expression of anti-apoptotic protein Bcl-2 (Figure 6 B,F) in both U_SH-SY5Y_ and D_SH-SY5Y_ cells. All three pre-treatments caused a significant reduction of the expression of the apoptotic proteins Bax and caspase 3 and an increase of the expression of the anti-apoptotic protein Bcl-2, with the exception of LD, in U_SH-SY5Y._ GSH and GSH-LD treatment caused a reduction of caspase 3 activity in U_SH-SY5Y_ cells (Figure 6D). Similarly, all three pre-treatments caused a significant reduction of the expression of caspase 3 (Figure 6G) and an increase of the expression Bcl-2 (Figure 6F) in D_U937_ cells, while only LD and GSH-LD were able to reduce the expression of Bax (Figure 6E). Only GSH-LD was able to reduce caspase 3 activity in D_SH-SY5Y_ cells (Figure 6H).

These data collectively indicate that GSH-LD more effectively protected cell death in H_2_O_2_ treated U_SH-SY5Y_ and D_SH-SY5Y_ cells.

### 3.4. Effect of GSH-LD on the U937 Cell Mediated Cytotoxic in SH-SY5Y Cells

**Figure 7:**
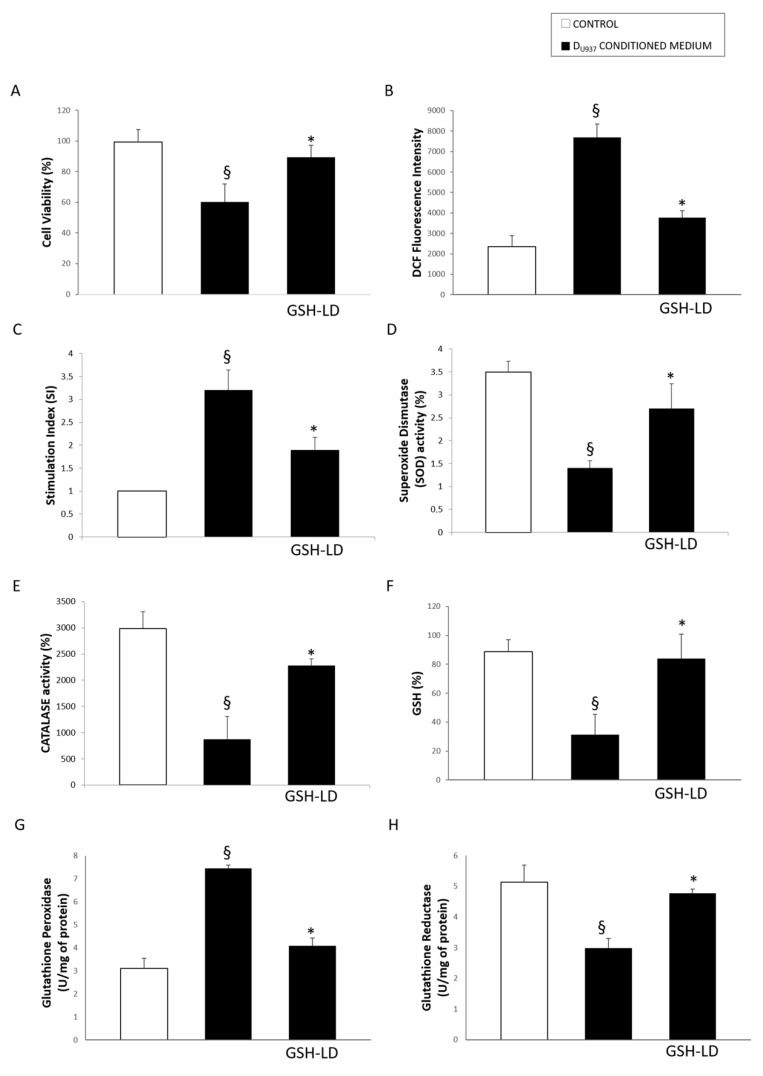
Effect of GSH-LD on D_SH-Sy5Y_ cells treated with conditioned medium obtained from activated D_U937_ cells. D_SH-Sy5Y_ cells pre-treated with GSH-LD (1 μM, 1 h) and exposed to conditioned medium obtained from activated D_U937_ cells. After 24 h were conducted: (**A**) MTT assay; (**B**) Reactive oxygen species (ROS) production measured by DCF (2′,7′-dichlorofluorescin diacetate) fluorescence; (**C**) Measurement of ROS levels using NBT (Nitroblue tetrazolium) assay expressed as stimulation index; (**D**) Superoxide Dismutase (SOD) activity (**E**) Catalase (CAT) activity; (**F**) GSH level; (**G**) Glutathione Peroxidase (GPx) and H) Glutathione Reductase (GR) assay. Values are mean ± SD, *n* = 5; ^§^ *p* < 0.05 vs. CTRL; * *p* < 0.05 vs. cells treated with conditioned medium.

The impact of resident-peripheral immune cells in the development of PD was investigated in an in vitro neurotoxicity model in which D_U937_ cells, imitating microglia cells, were activated with H_2_O_2_ (500 μM, 4 h), and their secretion was used as “conditioned medium” to induce toxicity in D_SH-SY5Y_ cells. The cytotoxic effect of conditioned medium obtained from activated D_U937_ cells on D_SH-SY5Y_ was evaluated by assessing cell viability, intracellular and extracellular ROS production, the cellular redox status (by measuring SOD and CAT, responsible for scavenging metabolites generated by free radicals) and GSH metabolism (by measuring the activity of GPx, which couples oxidation of GSH to detoxify H_2_O_2_, and GR, which reduces GSSG back to GSH, and GSH level). Clearly, the conditioned medium obtained from activated D_U937_ cells was able to induce a cytotoxic response in D_SH-SY5Y_ cells since it was causing a significant reduction of cell viability (Figure 7A), an increased ROS production (Figure 7B,C), reducting the activity of the antioxidant enxymes SOD (Figure 7D), CAT (Figure 7E), and GR (Figure 7H), increasing the activity of GPx (Figure 7G) and reducing GSH levels (Figure 7F).

Interestingly, the pretreatment of D_SH-SY5Y_ with GSH-LD (1 µM) provided a protection against D_U937_ conditioned medium mediated cytotoxicity. Indeed, GSH-LD was able to preserve cell viability, cellular redox status, GSH metabolism and prevent ROS formation.

Since kinase B (Akt)/phosphinositide 3-kinase (PI3K) is the canonical pathway involved in ROS-mediated apoptosis, the activation of this pathway was evaluated to investigate the mechanism involved in the anti-apoptotic effect of GSH-LD. Activated Akt can suppress cell apoptosis by inhibiting pro-apoptotic proteins such as caspase 3 and Bax and by activating anti-apoptotic proteins such as Bcl-2 [22,23]. Upstream of Akt, PI3K recruits and activates the phosphoinositide-dependent kinase, thereby activating Akt by phosphorylation [24,25].

**Figure 8:**
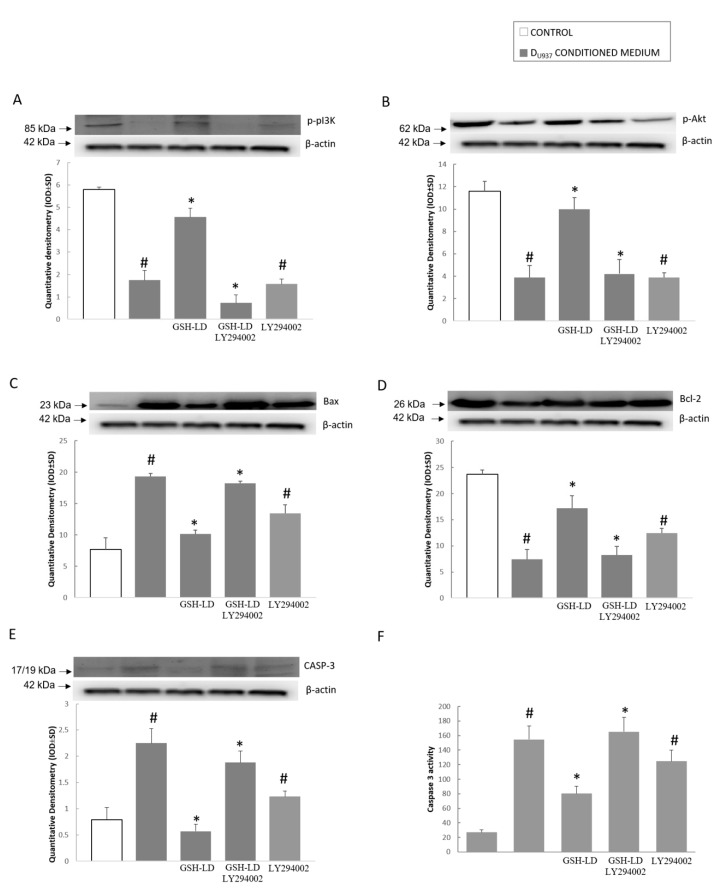
Effect of GSH-LD on phosphinositide 3-kinase/kinase B (PI3K/Akt) pathway in D_SH-Sy5Y_ cells treated with D_U937_ cells conditioned medium. D_SH-Sy5Y_ cells were preincubated with GSH-LD (1 μM) for 1 h, followed by treatment with D_U937_ cells conditioned medium. Representative image of Western blot experiments (top) and data analysis (bottom) showing expression of (**A**) p-PI3k; (**B**) p-Akt; (**C**) Bax; (**D**) Bcl-2; (**E**) Caspase 3 on total protein extracts of SH-SY5Y cell. Caspase 3 activity (**F**) was examined using the fluorogenic substrate Ac(N-acetyl)-DEVD-AMC (7-amino-4-methylcoumarin). LY294002 (a selective PI3K inhibitor) was used to evaluate the effect on the expression of related proteins in D_SH-Sy5Y_ cells after GSH-LD treatment. ß-actin was used as internal standard. The data are expressed as the mean ± SD of three independent experiments. ^#^ *p* < 0.05 vs. CTRL; * *p* < 0.05 vs. cells treated with conditioned medium.

The conditioned medium treatment obtained from H_2_O_2-_tretaed D_U937_ cells was able to inhibit the PI3K/Akt signaling pathway in D_SH-SY5Y_ cells as the phosphorylation of PI3K and Akt was reduced (Figure 8A,B). Moreover, the conditioned medium reduced Bcl-2 expression (Figure 8D) but elevated Bax and caspase 3 expression (Figure 8C,E) and caspase 3 activity (Figure 8F). The pre-treatment of D_SH-SY5Y_ with GSH-LD was able to restore the PI3K and Akt phosphorylation (Figure 8A,B) and prevent changes in Bax, Bcl-2 and caspase 3 expression (Figure 8C,D,E) and caspase 3 activity (Figure 8F). The PI3K inhibitor, LY294002 (20 μM), was able to reverse the effect of GSH-LD on the PI3K/Akt signaling pathway (Figure 8). In summary, these results demonstrate that GSH-LD inhibited conditioned medium-induced apoptosis in a PI3K/Akt-dependent manner in a neurotoxicity model in which D_U937_ cells were activated with H_2_O_2_ and their conditioned medium was used to induce toxicity to D_SH-SY5Y_ cells.

## 4. Discussion

Both human and animal studies indicate that the progressive and specific loss of dopaminergic neurons in the CNS is associated with a dysregulation of cellular oxidative status and plays an important role in the neuro-inflammatory process that contributes to the pathogenesis of PD [26]. The neuro-inflammation process is characterized by the presence of reactive astrocytes and activated microglia that produce cytokines, chemokines, prostaglandins, and reactive oxygen and nitrogen species (ROS/RNS), which then can be responsible for the disruption of the blood-brain barrier (BBB) [27]. The deregulation of the neuro-immune system may be contributing to the chronic nature of PD [28]. In addition to neuro-inflammation, the peripheral immune system is also implicated in the pathogenesis of PD [29]. During the progression of PD, the initial neuro-protective action guaranteed by microglia becomes toxic to dopaminergic neurons as a result of the overproduction of ROS/RNS and cytokines. In parallel to this process, microglia recruit peripheral immune cells to act on the brain, resulting in a dynamic cross-regulation of their respective phenotypes [30]. In this study, we used an experimental PD model that takes in account the interplay between the dopaminergic neurons and microglia, the pro-oxidant properties of LD deriving from autoxidative metabolism and the reduced GSH levels. We investigated the effect of the GSH-LD codrug, synthesized by our research group, in undifferentiated and differentiated lymphoma U-937 cell lines and SH-SY5Y dopaminergic neuroblastoma cell lines, respectively used as models to study the involvement of macrophages/microglia and dopaminergic neurons in PD pathogenesis [20]. The approach to use a reversible bioconjugate (GSH-LD) for treatment of neurodegenerative disease, is related to its ability to prolong plasma LD levels and striatal dopamine-concentration compared to an equimolar dose of LD, as previously described in the rat Parkinson’s model [9].

We studied the effect of GSH-LD on apoptosis and cellular oxidative stress, since both processes are considered strategic targets for the prevention of the neurodegenerative disease and for neuroprotective therapy.

GSH-LD showed an anti-apoptotic effect in H_2_O_2_ treated undifferentiated and differentiated monocytes and dopaminergic cells, although its impact is already effective at the lowest concentration used in neuronal cells, probably due to different expression and activity of receptors and/or to the involvement of different signaling pathways in the two cell types. It is noteworthy that the mismatch resulting between apoptotic process and markers of apoptosis such as caspase 3 activity, could suggest that in undifferentiated monocytes the LD treatments are carried out in an apoptosis independent-caspase fashion where the AIF (apoptosis inducing factor) is released from mitochondria and induces chromatin condensation [23,31]. In neuronal cells, differentiation seems to make a large difference to the significance of the protection against apoptosis. Considering that differentiated neuronal cells are more close to the human situation and GSH alone is just as protective against hydrogen peroxide cytotoxicity, the GSH-LD co-drug showed a more remarkable efficacy. GSH-LD treatment contrasted the reduction of Bcl-2 expression induced by H_2_O_2_ in monocytic and neuronal cells, underlining the essential role of Bcl-2 in the neuroprotection of GSH-LD against H_2_O_2_-induced apoptosis. This is consistent with the antioxidant function presented by Bcl-2 to prevent apoptosis as previously reported [32].

Several reports showed that microglia activation is an advantage under most conditions, but their hyperactivation may be detrimental to the nervous tissue since they generate inflammatory cytokines, which are potentially neurotoxic [33,34].

In the second part of the study we investigated the effect of GSH-LD in a neurotoxicity model in which neurotoxicity in differentiated SH-SY5Y was mediated by the conditioned medium obtained by H_2_O_2_-treated differentiated U937 cells. In this model, that mimics the interplay between microglia and neurons, the pretreatment of the dopaminergic SH-SY5Y cells with GSH-LD was able to protect them from cell death, delay apoptosis, prevent oxidative damage and restore cellular oxidative balance.

In our previous study, we demonstrated the antioxidant efficacy of GSH-LD codrug by measuring peripheral markers of oxidative stress such as SOD and GPx enzymes in rat plasma [9]. In the present study we observed that GSH-LD treatment prevented ROS intra- and extracellular production by restoring an antioxidant cellular system in activated neurons. GSH-LD treatment promoted the expression of pro-survival proteins and simultaneously inhibited pro-apoptotic proteins by triggering the PI3K/Akt-mediated signaling pathway. The crucial role of Akt as a cellular survival factor against oxidative stress was confirmed by using a PI3K inhibitor, LY294002, that was able to prevent the inhibitory effect GSH-LD on PI3K/Akt signaling pathways. We have demonstrated that the GSH-LD codrug, obtained by the binding of L-Dopa with GSH, offers advantages deriving from an additive effect of both molecules. The co-presence of GSH allows the recovery of GSH levels, which usually are reduced in PD, and also contrasts the pro-oxidant effect due to the presence of the catecholic moiety in LD.

In conclusion, despite LD administration being a standard treatment in PD, some reports have reported its neurotoxicity caused by the generation of oxygen/nitrogen reactive species [35]. In our study we have confirmed a marked pro-apoptotic effect of LD in undifferentiated and differentiated monocytic and neuronal cells, depending on the activation of molecules such as Bax and caspase 3. Moreover, we have shown that the GSH-LD prodrug offers advantages deriving from a synergistic effect of LD and GSH in a cellular model of PD.

We acknowledge that vitro models may not be clinically relevant, but our findings suggest that GSH-LD, due to antioxidant and anti-apoptotic effects against neuronal cell toxicity, could be a promising candidate for the treatment of PD. Certainly, the significance of our observations need to be validated in further studies investigating the neuroprotective effects of GSH-LD in PD in in vivo animal models and its ability to penetrate effectively the BBB.

## Supplementary Materials

The following are available online at https://www.mdpi.com/2076-3921/8/8/319/s1, Figure S1: U937 and SH-SY5Y differentiation selective markers.

## References

[1] Tofaris G.K., Spillantini M.G. (2005). Alpha-synuclein dysfunction in Lewy body diseases. Mov. Disord..

[2] Spencer J.P., Jenner A., Butler J., Aruoma O.I., Dexter D.T., Jenner P., Halliwell B. (1996). Evaluation of the pro-oxidant and antioxidant actions of L-Dopa and dopamine in vitro: Implications for Parkinson’s disease. Free Radic. Res..

[3] Elfawy H.A., Das B. (2019). Crosstalk between mitochondrial dysfunction, oxidative stress, and age related neurodegenerative disease: Etiologies and therapeutic strategies. Life Sci..

[4] Kausar S., Wang F., Cui H. (2018). The Role of Mitochondria in Reactive Oxygen Species Generation and Its Implications for Neurodegenerative Diseases. Cells.

[5] Schieber M., Chandel N.S. (2014). ROS function in redox signaling and oxidative stress. Curr. Biol..

[6] Gerfen C.R. (2003). D1 dopamine receptor supersensitivity in the dopamine-depleted striatum animal model of Parkinson’s disease. Neuroscientist.

[7] Gu F., Chauhan V., Chauhan A. (2015). Glutathione redox imbalance in brain disorders. Curr. Opin. Clin. Nutr. Metab. Care.

[8] Smeyne M., Smeyne R.J. (2013). Glutathione metabolism and Parkinson’s disease. Free Radic. Biol. Med..

[9] Pinnen F., Cacciatore I., Cornacchia C., Sozio P., Iannitelli A., Costa M., Pecci L., Nasuti C., Cantalamessa F., Di Stefano A. (2007). Synthesis and study of L-Dopa-glutathione codrugs as new anti-Parkinson agents with free radical scavenging properties. J. Med. Chem..

[10] Kovalevich J., Langford D. (2013). Considerations for the use of SH-SY5Y neuroblastoma cells in neurobiology. Methods Mol. Biol..

[11] Pesce M., Speranza L., Franceschelli S., Ialenti V., Patruno A., Febo M.A., De Lutiis M.A., Felaco M., Grilli A. (2011). Biological role of interleukin-1beta in defensive-aggressive behaviour. J. Biol. Regul. Homeost. Agents.

[12] Liberman A.C., Trias E., da Silva Chagas L., Trindade P., Dos Santos Pereira M., Refojo D., Hedin-Pereira C., Serfaty C.A. (2018). Neuroimmune and Inflammatory Signals in Complex Disorders of the Central Nervous System. Neuroimmunomodulation.

[13] Colombo C., Cosentino M., Marino F., Rasini E., Ossola M., Blandini F., Mangiagalli A., Samuele A., Ferrari M., Bombelli R. (2003). Dopaminergic modulation of apoptosis in human peripheral blood mononuclear cells: Possible relevance for Parkinson’s disese. Ann. N. Y. Acad. Sci..

[14] Patruno A., Franceschelli S., Pesce M., Maccallini C., Fantacuzzi M., Speranza L., Ferrone A., De Lutiis M.A., Ricciotti E., Amoroso R. (2012). Novel aminobenzyl-acetamidine derivative modulate the differential regulation of NOSs in LPS induced inflammatory response: Role of PI3K/Akt pathway. Biochim. Biophys. Acta.

[15] Klegeris A., McGeer P.L. (2000). Interaction of various intracellular signaling mechanisms involved in mononuclear phagocyte toxicity toward neuronal cells. J. Leukoc. Biol..

[16] Iarlori C., Gambi D., Lugaresi A., Patruno A., Felaco M., Salvatore M., Speranza L., Reale M. (2008). Reduction of free radicals in multiple sclerosis: Effect of glatiramer acetate (Copaxone). Mult. Scler..

[17] Franceschelli S., Pesce M., Ferrone A., Gatta D.M., Patruno A., De Lutiis M.A., Quiles J.L., Grilli A., Felaco M., Speranza L. (2017). Biological Effect of Licochalcone C on the Regulation of PI3K/Akt/eNOS and NF-κB/iNOS/NO Signaling Pathways in H9c2 Cells in Response to LPS Stimulation. Int. J. Mol. Sci..

[18] Franceschelli S., Pesce M., Ferrone A., Patruno A., Pasqualone L., Carlucci G., Ferrone V., Carlucci M., De Lutiis M.A., Grilli A. (2016). A Novel Biological Role of α-Mangostin in Modulating Inflammatory Response Through the Activation of SIRT-1 Signaling Pathway. J. Cell. Physiol..

[19] Speranza L., Franceschelli S., Pesce M., Vinciguerra I., De Lutiis M.A., Grilli A., Felaco M., Patruno A. (2008). Phosphodiesterase type-5 inhibitor and oxidative stress. Int. J. Immunopathol. Pharm..

[20] Cacciatore I., Marinelli L., Di Stefano A., Di Marco V., Orlando G., Gabriele M., Gatta D.M.P., Ferrone A., Franceschelli S., Speranza L. (2018). Chelating and antioxidant properties of l-Dopa containing tetrapeptide for the treatment of neurodegenerative diseases. Neuropeptides.

[21] Xiang J., Wan C., Guo R., Guo D. (2016). Is Hydrogen Peroxide a Suitable Apoptosis Inducer for All Cell Types?. Biomed. Res. Int..

[22] Redza-Dutordoir M., Averill-Bates D.A. (2016). Activation of apoptosis signalling pathways by reactive oxygen species. Biochim. Biophys. Acta.

[23] Kroemer G., Martin S.J. (2005). Caspase-independent cell death. Nat. Med..

[24] Kim J.H., Na H.J., Kim C.K., Kim J.Y., Ha K.S., Lee H., Chung H.T., Kwon H.J., Kwon Y.G., Kim Y.M. (2008). The non-provitamin A carotenoid, lutein, inhibits NF-kappaB-dependent gene expression through redox-based regulation of the phosphatidylinositol 3-kinase/PTEN/Akt and NF-kappaB-inducing kinase pathways: Role of H(2)O(2) in NF-kappaB activation. Free Radic. Biol. Med..

[25] Nakano N., Matsuda S., Ichimura M., Minami A., Ogino M., Murai T., Kitagishi Y. (2017). PI3K/AKT signaling mediated by G protein coupled receptors is involved in neurodegenerative Parkinson’s disease. Int. J. Mol. Med..

[26] Gorelenkova Miller O., Mieyal J.J. (2015). Sulfhydryl-mediated redox signaling in inflammation: Role in neurodegenerative diseases. Arch. Toxicol..

[27] Boyko A.A., Troyanova N.I., Kovalenko E.I., Sapozhnikov A.M. (2017). Similarity and Differences in Inflammation-Related Characteristics of the Peripheral Immune System of Patients with Parkinson’s and Alzheimer’s Diseases. Int. J. Mol. Sci..

[28] Ransohoff R.M., Perry V.H. (2009). Microglial physiology: Unique stimuli, specialized responses. Annu. Rev. Immunol..

[29] Wang Q., Liu Y., Zhou J. (2015). Neuroinflammation in Parkinson’s disease and its potential as therapeutic target. Transl. Neurodegener..

[30] Lull M.E., Block M.L. (2010). Microglial activation and chronic neurodegeneration. Neurotherapeutics.

[31] Cregan S.P., Dawson V.L., Slack R.S. (2004). Role of AIF in caspase-dependent and caspase-independent cell death. Oncogene.

[32] Hockenbery D.M., Oltvai Z.N., Yin X.M., Milliman C.L., Korsmeyer S.J. (1993). Bcl-2 functions in an antioxidant pathway to prevent apoptosis. Cell.

[33] Heyes M.P., Achim C.L., Wiley C.A., Major E.O., Saito K., Markey S.P. (1996). Human microglia convert l-tryptophan into the neurotoxin quinolinic acid. Biochem. J..

[34] Kingham P.J., Pocock J.M. (2001). Microglial secreted cathepsin B induces neuronal apoptosis. J. Neurochem..

[35] Shin K.S., Choi H.S., Zhao T.T., Suh K.H., Kwon I.H., Choi S.O., Lee M.K. (2013). Neurotoxic effects of berberine on long-term L-Dopa administration in 6-hydroxydopamine-lesioned rat model of Parkinson’s disease. Arch. Pharm. Res..

