# Supplementary figures and images for: Modulation of Apoptotic Cell Death and Neuroprotective Effects of Glutathione—L-Dopa Codrug Against H2O2-Induced Cellular Toxicity
Source: Antioxidants (Basel). 2019 Aug 19;8(8):319. doi: 10.3390/antiox8080319 (PMC6720001; doi:10.3390/antiox8080319)

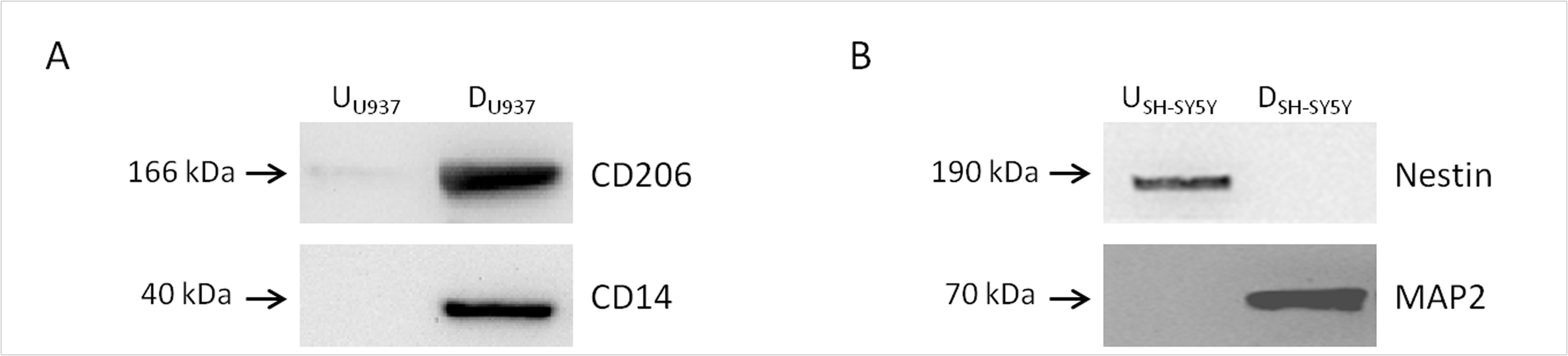

Supplement: Supplementary file 1 [file antioxidants-08-00319-s001.zip › antioxidants-555708-SI/antioxidants-555708-supplementary.TIF]
